# High Precision Feature Fast Extraction Strategy for Aircraft Attitude Sensor Fault Based on RepVGG and SENet Attention Mechanism

**DOI:** 10.3390/s22249662

**Published:** 2022-12-09

**Authors:** Zhen Jia, Kai Wang, Yang Li, Zhenbao Liu, Jian Qin, Qiqi Yang

**Affiliations:** 1 School of Mechanical and Electrical Engineering, Xi’an University of Architecture and Technology, Xi’an 710055, China; 2 School of Civil Aviation, Northwestern Polytechnical University, Xi’an 710072, China

**Keywords:** attitude sensor, fault diagnosis, attention mechanism, time-frequency signal, RepVGG

## Abstract

The attitude sensor of the aircraft can give feedback on the perceived flight attitude information to the input of the flight controller to realize the closed-loop control of the flight attitude. Therefore, the fault diagnosis of attitude sensors is crucial for the flight safety of aircraft, in view of the situation that the existing diagnosis methods fail to give consideration to both the diagnosis rate and the diagnosis accuracy. In this paper, a fast and high-precision fault diagnosis strategy for aircraft sensor is proposed. Specifically, the aircraft’s dynamics model and the attitude sensor’s fault model are built. The SENet attention mechanism is used to allocate weights for the collected time-domain fault signals and transformed time-frequency signals, and then inject the fused feature signals with weights into the RepVGG based on the convolutional neural network structure for deep feature mining and classification. Experimental results show that the proposed method can achieve good precision speed tradeoff.

## 1. Introduction

The complex structure, numerous equipment, system cross-linking and diverse flight environment of aircraft make it easy to have faults. The flight control system is the core system of the aircraft, in which the sensor is used to transmit the real-time measured aircraft flight state parameters to the flight control system. Therefore, the state of the sensor will directly affect the flight state [1,2]. Once a fault occurs, it will cause the sensor to transmit the wrong information to the flight control system, which may cause greater economic losses and even endanger people’s lives. Therefore, the diagnosis of aircraft sensors is essential to ensure aircraft flight safety [3,4].

The traditional fault diagnosis methods consist of mainly two types, one is based on signal analysis or artificial feature extraction [5,6], and the other is based on models [7,8]. Among them, a common application of the first method is to determine whether a fault occurs by designing a threshold value and comparing whether the signal reaches the threshold value [9]. Other methods based on manual feature extraction have also been widely studied. A fault diagnosis method based on signal decomposition and two-dimensional feature clustering is designed to diagnose battery status [10]. The data processing method of high-speed railway fault signal diagnosis based on MapReduce algorithm was designed [11]. Statistical method and wavelet packet decomposition method are used for feature extraction of vibration signal to identify the fault type of rotor [12].

The model-based method refers to establishing the model of the object to be diagnosed, and analyzing the situation when various faults occur by setting different types of faults in the model. Fault diagnosis is realized through the corresponding relationship between the output difference of the model in different faults and the fault type. A review of model-based fault diagnosis methods was published, focusing on fault detection and fault estimation [13]. N. Valceschini et al. proposed a model-based fault detection and isolation scheme for the transmission components of electro-mechanical actuators, which was applied to the drive of sliding doors [14]. In addition, a model-based battery fault diagnosis method is proposed, which is based on multiple equivalent circuit models [15]. In addition, Wang et al. established the equivalent circuit model of battery pack insulation fault diagnosis using the high fidelity unit model [16].

However, the two traditional methods mentioned above have some limitations, specifically , the method based on signal analysis diagnoses by manually selecting feature types, which is difficult to avoid the problem of insufficient representation of selected features [17]. The main problem of model-based method is that it requires high accuracy of the model, and it is no longer applicable when the object changes a little. With the development of machine learning and artificial intelligence technology, data-driven fault diagnosis method is very popular in recent years because of its advantages of automatically exploring the characteristics of signals and high applicability. More and more data driven diagnostic methods with higher accuracy have emerged [18,19,20,21,22,23]. A data driven method based on improved Elman neural network was proposed to realize the fast diagnosis of open circuit fault of IGBT [18]. Nicholas et al. [19] proposed a general robust data-driven scheme for fault detection, isolation and estimation of multiple sensor faults, and verified it with multiple flight data records. A fault diagnosis method based on Deep belief network (DBN) to generate local random graph to intuitively explain the fault action mechanism was proposed, which realized the diagnosis of different faults of air conditioner [20] Guo et al. [21] established a predictive model for photovoltaic power generation under normal conditions through clustering algorithm and long short-term memory neural network (LSTM), and used the predictive model to conduct quantitative fault diagnosis through transfer learning. In addition, some work related to fault diagnosis combines signal based and data-driven, or uses the transfer learning strategy [24,25,26,27] to achieve high-precision fault diagnosis results.

Different types of machine learning models have been targeted for development and used in data-driven diagnosis. However, there are still two problems in the processing of input data: the type of input signal data is single, which has no advantage in ensuring the integrity of data information; in a small amount of work considering multiple input signals, the importance of different signals is rarely considered, which is not conducive to subsequent feature extraction and fault classification. In addition, in view of the fact that most of the deep learning diagnosis models cannot give consideration to both time cost and computational efficiency, this paper also proposes a targeted scheme.

Specifically, the innovation points of this paper are as follows:

(i) The time sequence signal of aircraft attitude sensor is transformed into time-frequency domain, and the time-domain signal and time-frequency domain signal are taken as the feature mining object.
(ii) A signal representation weight analysis and allocation strategy is designed, and the representativeness of each channel of time-domain signal and time-frequency signal is analyzed by using Squeeze-and-excitation networks (SENet) attention mechanism.
(iii) A fast and high-precision diagnostic technology based on Re-parameterization visual geometry group (RepVGG) is proposed, which achieves a good diagnostic accuracy speed tradeoff.

The following text is arranged as follows: the relevant theories and methods are given in Section 2. Section 3 describes the experimental setup and the preparation process of the fault data set, including fault model building and data collection, experimental parameter settings, etc. The Section 4 presents the experimental results and discussions. Section 5 summarizes the full text.

## 2. Relevant Theories and Proposed Methods

### 2.1. RepVGG

The maturity of the “convolutional” neural network has made it a solution to many mainstream tasks. The commonly used convolutional neural network models in image classification include VGG-16 and ResNet. The performance of VGG network model will increase with its depth, which may lead to over fitting and gradient disappearance, and the accuracy will decline. ResNet model’s residual element can solve the gradient disappearance phenomenon well, but it is powerless for the common over fitting phenomenon of deep network. The multi-level branches in the residual structure in ResNet make the model difficult to implement. RepVGG network is a single path convolutional network architecture, which integrates the ideas of VGG and ResNet, and only adds 3 times. The 3-volume integration layer can also achieve simple and more efficient performance [28].

#### 2.1.1. RepVGG Block

**Figure 1:**
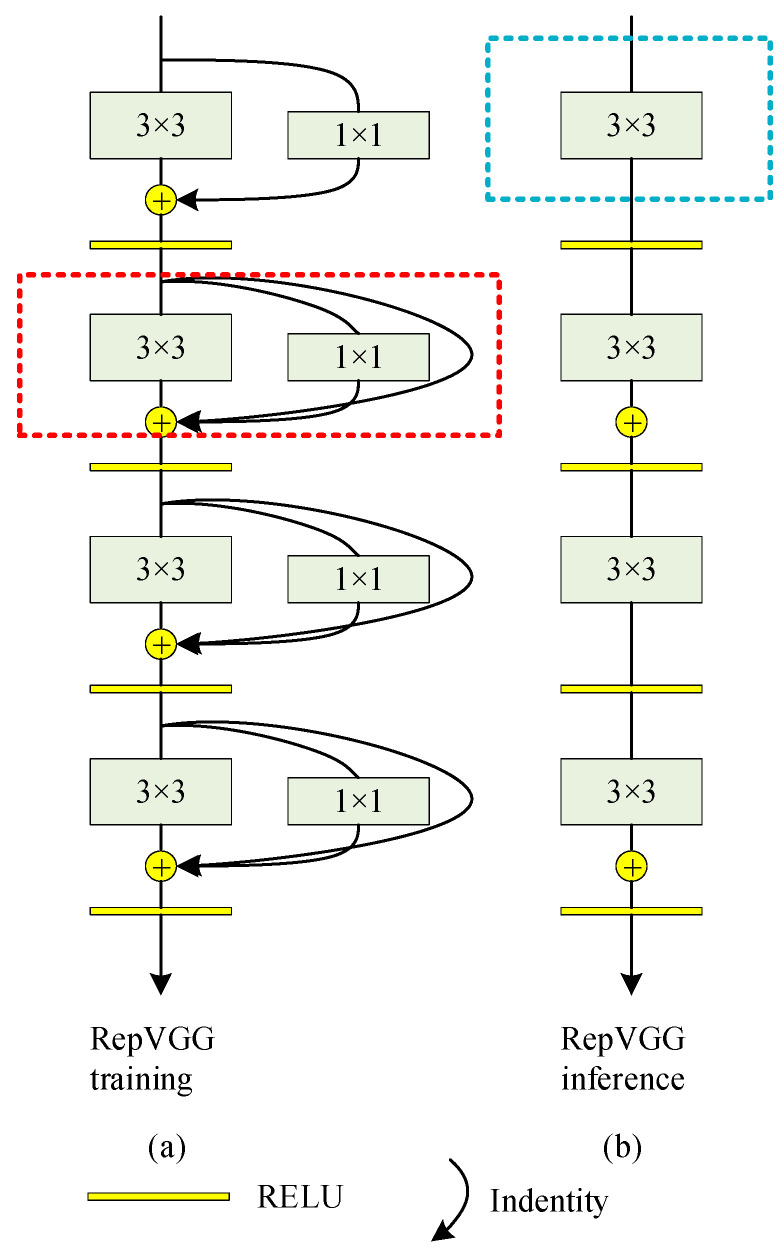
Schematic diagram of partial structure of RepVGG. (**a**) RepVGG training. (**b**) RepVGG inference.

As shown in Figure 1, RepVGG uses a multi branch model similar to ResNet style during training, and converts it into a single path model of VGG style during reasoning. Figure 1a shows the network structure used in RepVGG training, while Figure 1b is used in reasoning. Figure 1b shows the RepVGG network in the reasoning stage. The structure of the network is very simple. The whole network is composed of convolution with kernel size 3 × 3 and ReLu activation function, which is easy for model reasoning and acceleration.

RepVGG is formed by continuously stacking RepVGG Blocks. During the training, RepVGG Block paralleled three branches: a main branch with a convolution core size of 3 × 3, a shortcut branch with a convolution core size of 1 × 1, and a shortcut branch with only BN connected. Since the residual structure has multiple branches, it is equivalent to adding multiple gradient flow paths to the network. Such a network is trained, which is similar to training multiple networks and integrating multiple networks into one network. It is similar to the idea of model integration, which can improve the training effect of the network.

#### 2.1.2. Structural Reparameterization

**Figure 2:**
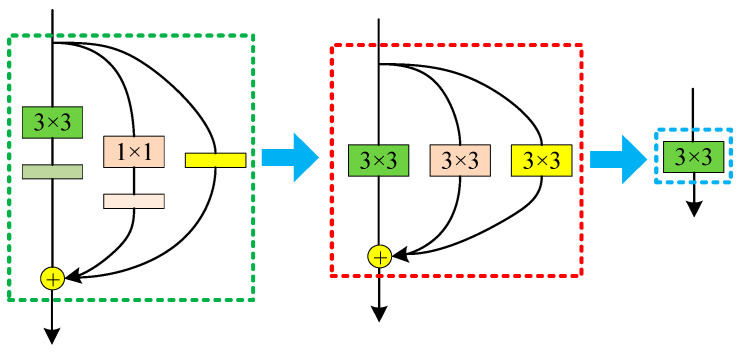
Schematic Diagram of Reparameterization.

RepVGG reparameterization transforms the multi branch structure in the training process into 3 × 3 convolution with deviation, which improves the reasoning speed of the network, reduces the network parameters and reduces the memory occupation. The process of reparameterization is shown in Figure 2, which includes four processes: merging Conv2d and BN; Convert 1 × 1 convolution to 3 × 3 convolution and BN to 3 × 3 convolution, and fuse multiple branches.

At the stage of merging 3 × 3 convolution layer and *BN* layer, the formula of convolution layer and *BN* layer is as follows:

(1) $$\begin{array}{l} \mathrm{Conv}(x)=W(x)\\ BN(x)=\gamma \times \frac{\left(x-\mu \right)}{\sqrt{\sigma_{i}^{2}+\epsilon }}+\beta \end{array}$$

where the input is *x*, the fusion of Conv into *BN* can be expressed as:

(2) $$BN(\mathrm{conv}(x))=\gamma \times \frac{W(x)-\mu }{\sqrt{\sigma_{i}^{2}+\epsilon }}+\beta =(\frac{\gamma \times W(x)}{\sqrt{\sigma_{i}^{2}+\epsilon }})+(\frac{\gamma \times W(x)}{\sqrt{\sigma_{i}^{2}+\epsilon }}+\beta )$$

The above formula can be regarded as the convolution layer incorporating *BN* operation, where $\sqrt{\sigma_{i}^{2}}$ is the variance of *BN* layer, *γ* Is the scale factor of *BN* layer, *β* Indicates the offset factor of *BN* layer. If the content in the first bracket of the above formula is regarded as $W^{\prime }$, and the content in the second bracket is regarded as $B^{\prime }$, then:

(3) $$W^{\prime }=\frac{\gamma \times W}{\sigma_{i}}$$

(4) $$B^{\prime }=\beta -\frac{\gamma \times \mu }{\sigma_{i}^{}}$$

Finally, it can be rewritten as:

(5) $$BN(\mathrm{Conv}(x))=W^{\prime }(x)+B^{\prime }(x)$$

When converting 1 × 1 convolution to 3 × 3 convolution form, take a convolution core in 1 × 1 convolution layer as an example, just add a circle of zeros around the original convolution core weight, which becomes a 3 × 3 convolution layer. Note that in order to ensure that the height and width of the input/output feature map remain unchanged, the padding is usually set to 1. Finally, the above convolution layer and BN layer can be fused.

When converting BN to 3 × 3 convolution, as there is no convolution layer for branches with only BN, a 3 × 3 convolution layer needs to be constructed first, and the convolution layer only carries out identity mapping, that is, the input and output characteristic maps remain unchanged. With this convolution layer, BN layer can be converted into 3 × 3 convolution.

Finally, multi branch fusion is carried out. The process of merging is relatively simple. The parameters of the three convolution layers are added together. In this step, the weight W and offset B of all branches are superposed to obtain a fused 3 × 3 Convolutional network layer.

### 2.2. SENet Attention Mechanism

Convolution often focuses on the fusion of scale information in space. Through the introduction of an attention mechanism, SENet focuses on the connection between different channels, so that it can learn the importance of each channel feature [29]. For the fault classification task in this paper, an attention mechanism is introduced to improve the attention of different characteristic channels of input signals. The SE module contains two operations: Squeeze and Exception; the global characteristics of each trace in the feature map can be obtained by the Squeeze operation. The relationship between channels can be learned through the Exception, and the weights between different channels can be obtained.

**Figure 3:**
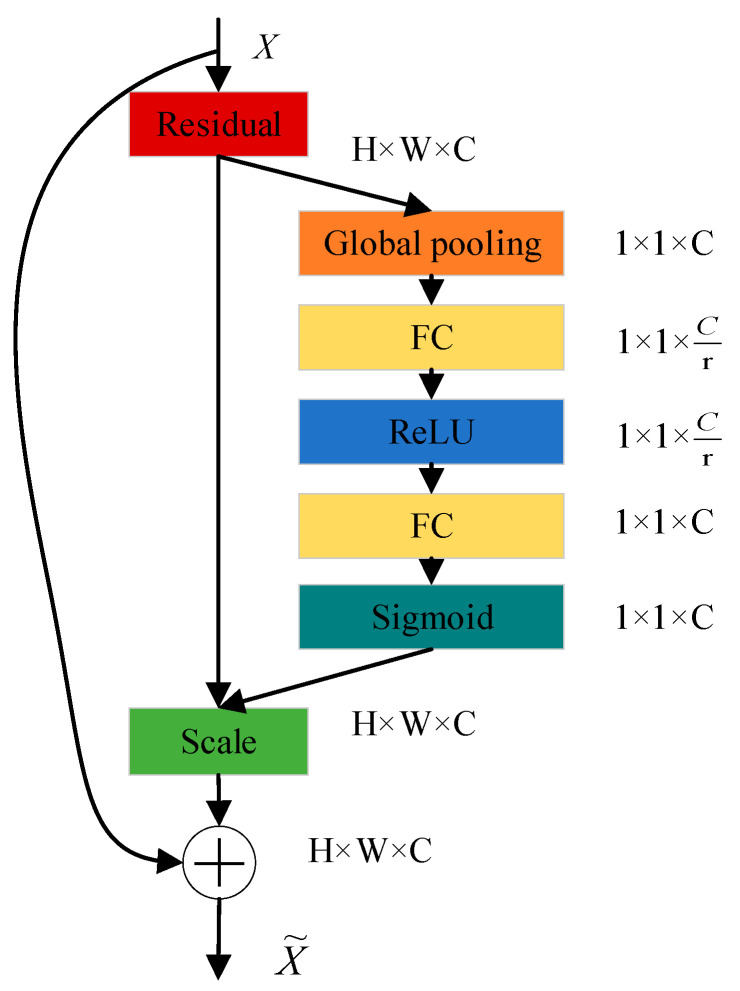
Structure of SENet.

Its implementation is shown in Figure 3. The input feature layer is pooled globally. Then two full connections are made. The number of fully connected neurons in the first time is less, and the number of fully connected neurons in the second time is the same as the input feature layer. A ReLU layer is set between two full connections. Then, another sigmoid is performed to fix the value between 0–1. At this time, the weight value of each channel of the input feature layer is obtained. Finally, the weighted feature layer can be obtained by multiplying this weight value by the original input feature layer.

### 2.3. The Proposed Strategy

**Figure 4:**
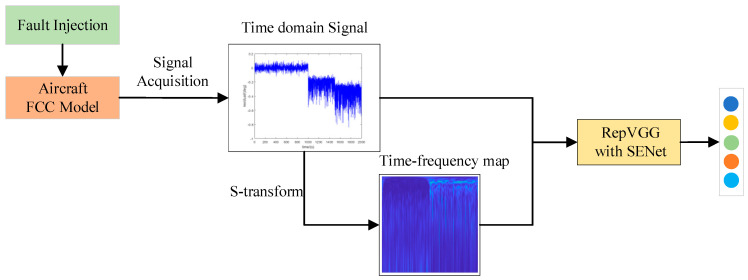
Flowchart of the algorithm proposed in this paper.

In order to fully exploit the characteristics of sensor fault signals, the fault diagnosis strategy shown in Figure 4 is proposed in this paper. First, different faults are injected into the flight control system model of the aircraft, and then the signals under the fault state are collected. The time-frequency characteristic diagram is obtained by processing one-dimensional time-domain residual signal through S-transform. The one-dimensional time-domain residual signal is sliced and stacked into a 50 × 50 × 1 format. As the first channel data, the data size of the RGB three channels of time-frequency characteristic map is 50 × 50 × 3. The data of these four channels 50 × 50 × 4 are used as the processing data of the subsequent SENet attention mechanism and RepVGG. Because RepVGG only obtains features in spatial dimension, SENet module is integrated into RepVGG to obtain feature association between different channels. The proposed diagnostic algorithm consists of two parts: training and testing. The training part is to learn the model’s parameters by using the training data set, and the testing part is to test the effect of the proposed model by using the testing data set.

Add SE attention module to the 3 × 3 channel of RepVGG, and the feature map with size H × W × C is obtained after the convolution kernel operation (in the fault diagnosis task of this paper, the value of parameter C is 4). At this time, the convolution is only the characteristic diagram obtained by spatial operation, and there is no relationship between each channel; The 2D feature *p_c_* of each channel is mapped to the global feature *f_c_* through a global average pooling, and the formula is as follows:

(6) $$f_{c}=F_{sq}\left(p_{c}\right)=\frac{1}{h\times h}\sum_{i=1}^{h}\sum_{j=1}^{h}p_{c}(i,j),f\in R^{C}$$

Then, two Fully connected (FC) layers are used, one to reduce the dimension characteristics, and the other to upgrade back to the original dimension. Finally, the normalized weight is obtained through Sigmoid, and the formula is as follows:

(7) $$s=F_{ex}(f,W)=\sigma (g(f,W))=\sigma \left(W_{2}\mathrm{ReLU}\left(W_{1}z\right)\right)$$

(8) $$W_{1}\in R^{\frac{c}{r}\times C},W_{2}\in R^{\frac{c}{r}\times C}$$

Finally, the weight s obtained is weighted to each characteristic channel *f_c_*. This allows important channels to gain greater attention and ensure the accuracy of classification.

## 3. Experiment Setup and Data Set Preparation

### 3.1. Establishment of Fault Model

**Figure 5:**
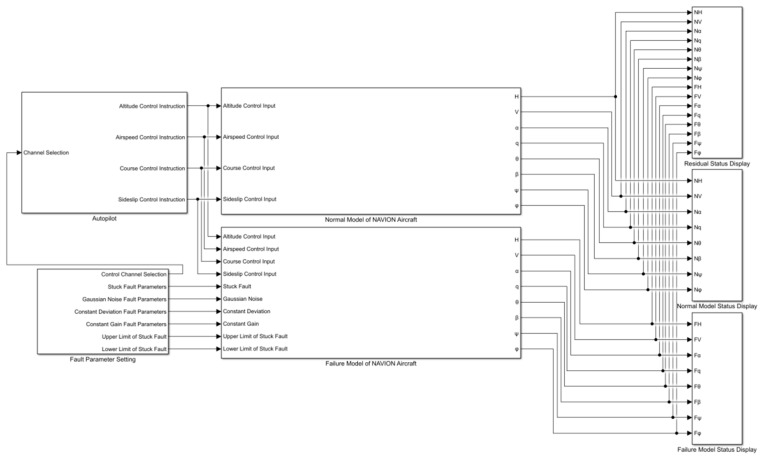
Simulation diagram of attitude sensor fault.

Navion aircraft model is built in this paper, and different fault types of its attitude sensor are set. Navion aircraft model is a navigation aircraft model. By the end of 1947, more than 1100 aircraft of this type had been produced in the United States. The aircraft has a total length of 8.38 m, a wingspan of 10.19 m, a height of 2.65 m, a maximum takeoff weight of 1338 kg, a maximum flight speed of 260 km per hour, and a maximum range of 1120 km. The aircraft was once designated as a training aircraft of the US Air Force. Today, there are still a large number of such aircraft in civilian use. In this paper, according to the published aerodynamic parameters of Navion aircraft, the first order Taylor expansion method is used to linearize the small disturbance equation of fixed wing aircraft at a certain equilibrium point, and the linearized model of Navion aircraft is obtained. As shown in Figure 5, a fault signal generation model is designed, including the normal sensor sensing part and the fault sensor sensing part. The input control signal is input into the control model of the aircraft. The attitude sensor in the normal attitude frame can correctly perceive the attitude information of the Unmanned aerial vehicles (UAV), while the attitude sensor in the fault attitude frame cannot correctly perceive the attitude information.

Among them, the attitude sensor in the normal attitude frame can correctly perceive the attitude information of the UAV, while the attitude sensor in the fault attitude frame cannot correctly perceive the attitude information. After the sensor fault model is established, the attitude information is measured and the fault output is obtained. Then calculate the residual of normal sensor data and fault sensor data, and use the residual time series signal as the data processed by the subsequent fault diagnosis model. The fault type settings are shown in Table 1, which contains the fault manifestations and corresponding labels. Taking the pitch angle sensor of an aircraft as an example, four common faults are set, including jamming, lateral gain, lateral deviation and excessive noise. In addition, if the fault free state is regarded as a special fault state, there are five fault types in total.

### 3.2. Acquisition of Fault Data

**Figure 6:**
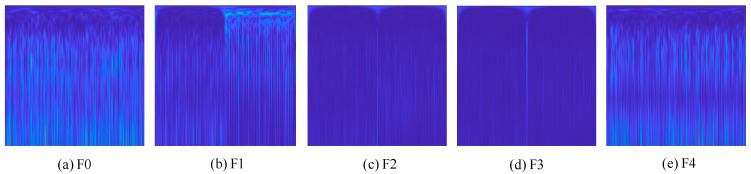
Signal diagrams for five different types of faults. (**a**) F0. (**b**) F1. (**c**) F2. (**d**) F3. (**e**) F4.

Set the corresponding input control signal to change within a certain angle range, collect the residual signals of various faults, the length of each residual signal is 2500, and rearrange them into a 50 × 50 format. In addition, the time-frequency diagram obtained by S-transform is also cut to 50 × 50 size, and 50 × 50 × 3 data obtained by extracting RGB three channels of color time-frequency diagram is used as the input of SENet attention mechanism model. Figure 6 shows the time-frequency diagram of different fault types after S transformation.

In order to explore the influence of different data sets on the fault diagnosis accuracy of the algorithm proposed in this paper, five data sets with different sizes are set. Table 2 lists the size information of training set, verification set and test set for each fault type.

### 3.3. Basic Parameter Settings of the Proposed Method

The parameters of the proposed method are shown in Table 3, where the kernel size is the size of the convolution kernel; Padding is the matrix filling value, that is, the filling is added to all four sides input, and the default value is 0; padding_ Mode is the matrix filling mode, and the default value is ‘zero’; num_ Blocks is the number of modules, that is, the number of sub modules in different stages; num_ Classes is the number of fault classifications, which is set as five fault states; width_ Multiplier is the stage multiplication coefficient, that is, the different coefficients multiplied at different stages; Groups is the number of input channel groups, that is, the number of blocked connections from the input channel to the output channel. The default value is 1; Street is the convolution step, and the default value is 1; The division is the expansion flag bit, that is, the spacing between kernel elements. The default value is 1; Bias adds a learnable deviation to the output. This parameter is a Boolean value. If it is true, a learnable deviation is added to the output, indicating that the parameters learned in the backward feedback are applied.

## 4. Experimental Results and Discussion

The experiments are conducted with Python 3.9, CUDA 11.6 and Pytorch 1.12.1 libraries on Windows11 operation system. The key experimental hardware configurations are NVIDIA GeForce RTX 3060 Laptop GPU with 6 GB memory and 12th Gen Intel(R) Core(TM) i9-12900H 2.50 GHz CPU with 16GB memory.

Accuracy is used to evaluate the diagnostic performance. Two indexes are as follows.

(9) $$Accuracy=\frac{N_{cp}}{N_{cp}+N_{wp}}$$

where *N_cp_* represents the number of cases whose label is correctly predicted, *N_wp_* refers the number of cases whose label is wrongly predicted.

### 4.1. Effect of Data Set on Diagnosis Results

The average precision and average training time are selected as the evaluation indicators of this paper to study the fault diagnosis performance of the method proposed in this paper under different data sets. The results are shown in Table 4.

Table 4 shows that: (i) As the size of the dataset increases (from dataset 1 to dataset 5), the model training time of the proposed algorithm becomes longer and longer. (ii) As far as the average precision is concerned, the precision has reached more than 99% in the case of data volume shown in dataset 3. Later, as the dataset continues to grow, for example, when it changes to dataset 5, compared with dataset 3, the average accuracy of the algorithm is only improved by 0.14%. According to Table 4, for the smallest dataset 1, its accuracy is the lowest, while for the medium dataset 3, the proposed algorithm has reached a satisfied accuracy. Therefore, we have added a Table 5 to list the accuracy of each fault type of a test in detail under the conditions of datasets 1 and 3.

As can be seen from Table 5, the corresponding classification accuracy of each fault label in dataset 3 is generally higher than that in dataset 1. In addition, in dataset 3, the number of tests for each fault type is 120, and the number of the wrong classification is less than 2.

**Figure 7:**
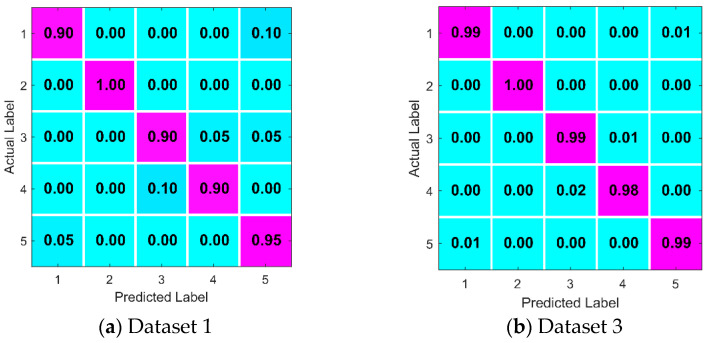
Confusion matrix under different data sets.

In order to better show the classification of the algorithm proposed in this paper under different data. The confusion matrix of dataset 1 and dataset 3 in an experiment is shown in Figure 7.

**Figure 8:**
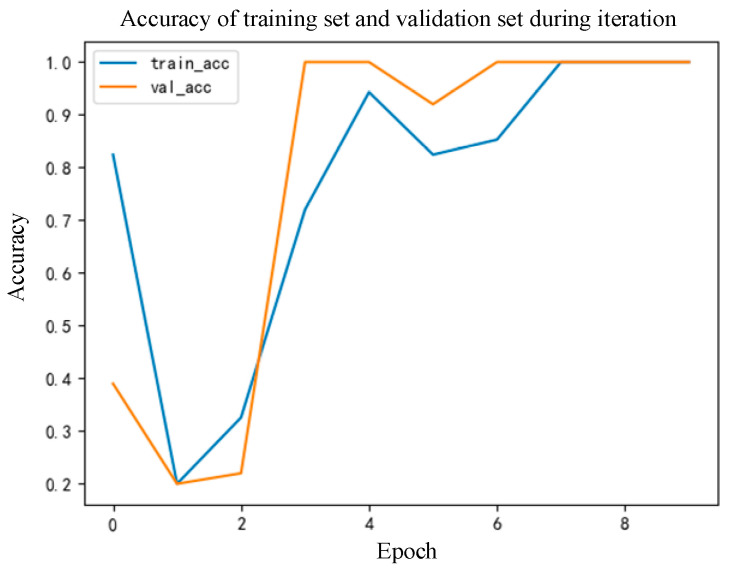
Iteration diagram of the accuracy of the algorithm proposed in this paper in the case of dataset 3.

It can be seen from Figure 7 that the misclassification of the proposed algorithm in dataset 3 is obviously better than that in dataset 1. Further, the model training process in the case of dataset 3 is shown in Figure 8.

It can be seen from Figure 8 that the training and verification stages of the model of the proposed diagnostic algorithm achieve the best accuracy at the 7th Epoch. In addition, the convergence speed of the model is relatively fast.

### 4.2. Ablation Experiment

In order to explore the role of each module of the method proposed in this paper. Ablation experiment was set up to conduct ablation research by deleting each module from the proposed method. (we conduct ablation studies by removing the identity and/or 1 × 1 branch from every block of RepVGG-B0.) Specifically, as shown in Table 6, respectively cancel the S transform module to use only the time domain signal (No. 1), cancel the time domain signal to use only the time-frequency domain signal (No. 2), cancel the SENet module (No. 3), and simultaneously cancel the S transform module and the SENet module (No. 4). The fault diagnosis accuracy under each condition is listed in this table.

Table 6 shows that the performance is the best when the SENet module is canceled, reaching 92.37%. Secondly, the performance is the second best when only time-frequency signals are used, reaching 86.35%. When the S-transform module is cancelled and only the time-domain signal is used, the corresponding diagnostic accuracy ranks third, only 80.68%. The performance is the worst when S-transform module and SENet module are canceled at the same time, which is only 62.75%. It can be seen from the results that the signal processed by the diagnosis strategy has the greatest impact on the diagnosis performance, and it is very important to obtain time-frequency diagram signal through S-transform. Secondly, the SENet module shows some advantages in data preprocessing, which provides a better basis for RepVGG to mine effective features. In addition, by comparing No. 1 and No. 2, it can be seen that compared with the timing signal, the time-frequency map obtained by using S-transform can better reflect the characteristics of the object.

## 5. Conclusions

In this paper, a fault diagnosis method for aircraft attitude sensor is proposed. Research shows that sensor residual signals can reflect the difference of various faults and can be used as a diagnostic signal. One dimension time-domain signals and two-dimensional time-frequency domain features are processed by SENet attention mechanism, and key feature categories are enhanced by high weight. Subsequently, the depth RepVGG is used to conduct in-depth feature mining and achieve a fast and high accuracy diagnosis effect. Therefore, the SENet attention mechanism is an effective feature importance ranking scheme, which realizes the weight division of signal categories while ensuring the integrity of diagnostic signals. In addition, RepVGG has also been proven to be a potential fault diagnosis algorithm, with obvious advantages in diagnosis speed while ensuring accuracy.

## Figures and Tables

**Table 1:** Setting of fault types.

Fault Type	Fault Manifestation	Fault Label
No fault	The fault free state represents the health state and is regarded as a special fault	F0
Stuck	The measured value of the sensor output deviates from the normal value and reaches a stuck position	F1
Constant gain	The measured value of the sensor output maintains a constant proportion to the normal output value	F2
Constant deviation	The measured value of the sensor output deviates from the normal value and keeps the deviation constant	F3
Excessive noise	The measured value of the sensor output contains large noise	F4

**Table 2:** Different data set size for each fault.

Data Set	Number of Training Sets	Number of Validation Sets	Number of Test Sets
1	140	20	40
2	280	40	80
3	420	60	120
4	700	100	200
5	980	140	280

**Table 3:** Size of the dataset.

Parameter	Value
kernel size	3
padding	0
padding_mode	‘zeros’
num_blocks	[2, 4, 14, 1]
num_classes	5
width_multiplier	[0.75, 0.75, 0.75, 2.5]
groups	1
stride	1
dilation	1
bias	True

**Table 4:** Diagnostic performance of the proposed method under different data sets.

Different Datasets	Dataset 1	Dataset 2	Dataset 3	Dataset 4	Dataset 5
Average accuracy (%)	93.45%	96.10%	99.28%	99.37%	99.42%
Average training time (s)	356.08	693.76	1019.73	1754.73	2390.49

**Table 5:** Accuracy detail presentation of data sets 1 and 3 in a test.

Dataset 1		Dataset 3	
Fault code	Accuracy (%)	Fault code	Accuracy (%)
F0	92.50	F0	98.33
F1	100.00	F1	99.17
F2	90.00	F2	100.00
F3	85.00	F3	98.33
F4	90.00	F4	99.17
Average accuracy	91.50	Average accuracy	99.00

**Table 6:** Fault diagnosis accuracy is tested in ablation study.

No.	Time Domain Signal	S-Transform	SENet	Average Accuracy
1	√		√	80.68%
2		√	√	86.35%
3	√	√		92.37%
4	√			62.75%

Note: The modules marked with √ in the table are reserved in the model.

## Data Availability

Data available on request due to restrictions, e.g., privacy or ethical.
